# Compositional Differences of Greek Cheeses of Limited Production

**DOI:** 10.3390/foods12122426

**Published:** 2023-06-20

**Authors:** Eleni C. Pappa, Efthymia Kondyli, Athanasios C. Pappas, Elisavet Giamouri, Aikaterini Sarri, Alexandros Mavrommatis, Evangelos Zoidis, Lida Papalamprou, Panagiotis Simitzis, Michael Goliomytis, Eleni Tsiplakou, Constantinos A. Georgiou

**Affiliations:** 1 Dairy Research Department, Institute of Technology of Agricultural Products, Hellenic Agricultural Organization-DIMITRA, Ethnikis Antistaseos 3, Katsikas, 45221 Ioannina, Greece; pappa.eleni@yahoo.gr (E.C.P.); kondyliefi@gmail.com (E.K.); 2 Laboratory of Nutritional Physiology and Feeding, Faculty of Animal Science, Agricultural University of Athens, 11855 Athens, Greece; egiamouri@aua.gr (E.G.); mavrommatis@aua.gr (A.M.); ezoidis@aua.gr (E.Z.); eltsiplakou@aua.gr (E.T.); 3 Laboratory of Animal Breeding and Husbandry, Department of Animal Science, Agricultural University of Athens, 75 Iera Odos, 11855 Athens, Greece; stud218084@aua.gr (A.S.); pansimitzis@aua.gr (P.S.); mgolio@aua.gr (M.G.); 4 Chemistry Laboratory, Department of Food Science and Human Nutrition, Agricultural University of Athens, 75 Iera Odos, 11855 Athens, Greece; lidapap@aua.gr (L.P.); cag@aua.gr (C.A.G.); 5 FoodomicsGR Research Infrastructure, Agricultural University of Athens, 75 Iera Odos, 11855 Athens, Greece

**Keywords:** cheesemaking, Greece, protected designation of origin, raw milk, ripening, traditional

## Abstract

Greece has a long tradition in cheesemaking, with 22 cheeses registered as protected designation of origin (PDO), 1 as protected geographical indication (PGI), and 1 applied for PGI. Several other cheeses are produced locally without any registration, which significantly contribute to the local economy. The present study investigated the composition (moisture, fat, salt, ash, and protein content), color parameters, and oxidative stability of cheeses that do not have a PDO/PGI certification, purchased from a Greek market. Milk and cheese types were correctly assigned for 62.8 and 82.1 % of samples, respectively, through discriminant analysis. The most important factors for milk type discrimination were L, a and b color attributes, salt, ash, fat-in-dry-matter, moisture-in-non-fat-substance, salt-in-moisture, and malondialdehyde contents, whereas a and b, and moisture, ash, fat, moisture-in-non-fat substance contents, and pH were the most influential characteristics for sample discrimination according to cheese type. A plausible explanation may be the differences in milk chemical composition between three animal species, namely cows, sheep, and goats and for the manufacture procedure and ripening. This is the very first report on the proximate analysis of these, largely ignored, chesses aiming to simulate interest for further study and production valorization.

## 1. Introduction

Greece has a long tradition in cheesemaking resulting in the production of many different cheeses. Twenty-two of them (Feta, Formaella Arachovas Parnassou, Ladotyri Mytilinis, Pichtogalo Chanion, Sfela, Manouri, Kefalograviera, Batzos, Anevato, Kopanisti, Xinomizithra Kritis, Kasseri, Katiki Domokou, Xigalo Siteias, Kalathaki Limnou, San Mihali, Graviera Agrafon, Arseniko Naxou, Metsovone, Arseniko Naxou, Graviera Kritis, and Graviera Naxou) have a protected denomination of origin (PDO) status and one (Krasotyri Ko/Tyri tis Possias) possesses a protected geographical indication (PGI) status while one (Kashkaval Pindou) has applied for PDI [1]. These cheeses fulfill certain, specific conditions, on the one hand, allowing consumers to make the best choice and, on the other hand, permitting the easier identification of these products on the market so as to facilitate checks [2]. The total production quantities of PDO/PGI Greek cheeses were 148,692 tonnes, for the year 2021 [3]; therefore, their manufacture plays an important role in the national economy of the country.

However, apart from the production of PDO/PGI cheeses, there are many others that are manufactured in Greece without being registered as PDO or PGI. In the year 2021, the production of Greek cheeses without a PGI/PDO status were 41,769 tonnes soft, 23,473 tonnes hard/semi hard, and 23,891 tonnes whey cheeses [3]. These quantities play an important role in the economy of their production places. Traditions and the local environment of the production place, the specific technological parameters, and the empirical approaches of the cheesemakers affect their identity. Some of these cheeses are produced widely and their characteristics are defined by the specific manufacture details that are applied during their production [4]. A few others are manufactured locally, even sometimes at a house, in limited quantities, using traditional facilities. Many of them remain unknown to the Greek population, let alone Europeans, and sometimes are only known in the regions they are made. As they sometimes exhibit a great variability, it is necessary to standardize their production and enhance their quality in accordance with safety regulations [5,6,7].

Nowadays, there is a growing demand from consumers for local cheeses with an assured quality. Many consumers would like to know more about the cheese as a product, the farming methods and practices applied, showing an understanding of the seasonality of production. Many producers of local cheeses promote seasonal products that are often linked with the maintenance of traditional animal breeds. These cheeses, which are manufactured regionally, can support the preservation of cultural heritage, and develop a sense of pride and belonging in the production area. It is known that local food systems can have multiple and broad ranging economic, environmental, and community development benefits. Local food systems can also diversify the rural economy, while a strong local food sector can encourage tourist activity by increasing the cultural identity of an area based around its local products and ensure the sustainability of local businesses [8].

The present study was part of a bigger project on the physicochemical properties, fatty acid, and elemental profile of PDO and non-PDO Greek cheeses. Previously, the physicochemical properties, fatty acid, and elemental profile of PDO Greek cheeses were studied [9,10]. During the design of the present study, the following issues were taken into consideration: (a) the necessity to survey a Greek market for local, less popular, non PDO cheeses available to consumers; (b) the necessity to analyze these local cheeses to highlight any compositional differences that may be present; and (c) if cheeses exposed to natural and artificial light throughout processing, packaging, and distribution are likely to be subjected to peroxidation [11]. Therefore, the aim of the present work was to characterize commercial non-PDO and non-PGI cheeses produced in Greece by determining their composition, color, and oxidative stability in order to establish their profile, as some of them, to the best of our knowledge, are studied for the first time and make them more familiar to consumers.

## 2. Materials and Methods

### 2.1. Sample Collection

**Figure 1:**
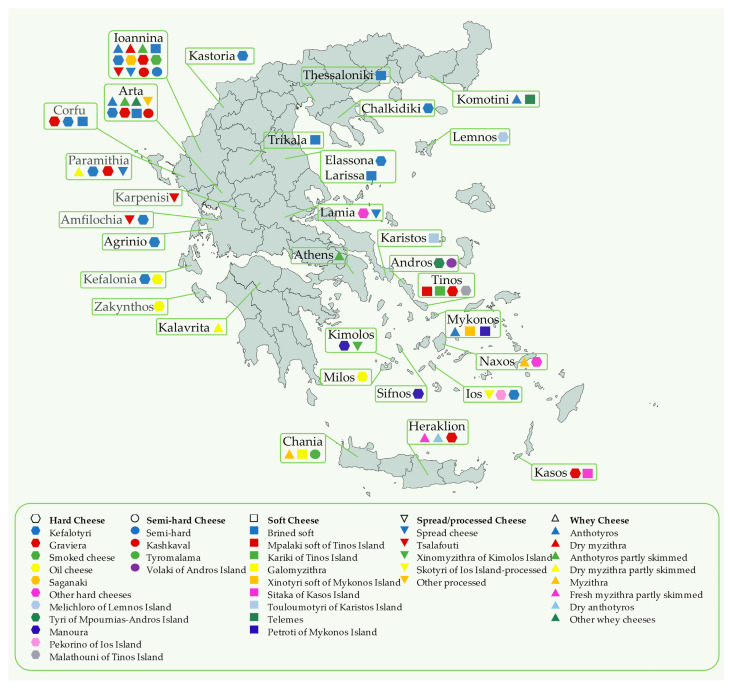
Map of Greece showing the regions for the cheese sample collection.

A total of 102 cheese samples were purchased from local stores and supermarkets from different parts of Greece, from January to May 2022. Samples (200–500 g) were milled, homogenized, or macerated to obtain a uniform material. The samples were analyzed immediately or when necessary, and they were stored at −32 °C until analysis. A map of collected samples from different regions of Greece is presented in Figure 1.

### 2.2. Composition and Physicochemical Characteristics

The composition (i.e., moisture, fat, salt, and protein), pH, moisture in non-fat substance (MNFS), and fat in dry matter (FDM) were determined as described by Danezis et al. [9]. The salt in moisture (SM) content was calculated using Equation (1):

(1) $$SM\;\%=salt\;\%\times \frac{100}{moisture\;\%}$$

For the determination of the ash content [12], porcelain dishes were placed in the oven for at least 2 h and then kept at room temperature. Each porcelain dish was then weighed, received 1 g of cheese, and then heated at 550 ℃ for 5–6 h. After reaching room temperature and weighting, the ash was determined as result of Equation (2):

(2) $$Ash\;\%=\frac{(W_{2}-W_{0})\;\%}{\left(W_{1}-W_{0}\right)}$$

*W*_0_ = weight of empty dish;

*W*_1_ = weight of dish with sample;

*W*_2_ = weight of dish and sample after incineration.

Cheese color was measured with a Miniscan XE chromameter (HunterLab, Reston, VA, USA) set on the L* (lightness), a* (redness), and b* (yellowness) system. The instrument was calibrated with a white and a black tile using illuminant D65 with 0° viewing.

Cheese oxidative stability was assessed on the basis of the malondialdehyde (MDA) content, which is a secondary product that originated from the hydrolysis of lipid hydroperoxides. In the present study, MDA levels were determined using a selective third-order derivative spectrophotometric method. In brief, 2 g of each sample was homogenized (Unidrive × 1000, CAT, M. Zipperer GmbH, Germany) in the presence of 5 mL butylated hydroxytoluene in hexane (8 g/L) and 8 mL aqueous trichloroacetic acid (TCA) (50 g/L), and the mixture was centrifuged for 5 min at 3000× *g*. The top hexane layer was discarded and a 2.5 mL aliquot from the bottom layer was mixed with 1.5 mL of aqueous 2-thiobarbituric acid (8 g/L) to be further incubated at 70 °C for 30 min. Following incubation, the mixture was cooled under tap water and submitted to third-order derivative (3D) spectrophotometry (Hitachi U3010 Spectrophotometer, Hitachi High-Technologies Corporation, Japan) in the range of 500–550 nm. The concentration of MDA (ng/g wet tissue) in the samples was determined as the height of the third-order derivative peak at 521.5 nm by referring to the standard calibration curve prepared using 1,1,3,3-tetraethoxypropane, the malondialdehyde precursor [13]. In all cases, duplicate cheese samples were analyzed.

### 2.3. Statistical Analysis

Data were subjected to analysis of variance with the type of cheese, type of milk, and the origin (island or mainland Greece) as fixed factors. Multiple comparisons were applied with Bonferroni adjustment. A discriminant analysis was applied to examine whether the samples could be distinguished according to the type of cheese and type of milk, based on their composition and physicochemical characteristics. A stepwise discriminant analysis was performed in order to reveal elements that were mainly responsible for the observed discrimination. Cheeses with only one sample per milk type were excluded from the discriminant analysis and analysis of variance. The level of significance, *p*-value, was set at 0.05. Statistical analysis was performed using SAS software [14]. Data composition and physicochemical characteristics of the cheeses are shown as mean ± Standard Error (S.E.) and the effect of main factors on the composition and physicochemical characteristics of cheeses are presented as least square means (L.S.M.) ± S.E.

## 3. Results and Discussion

### 3.1. Composition and Physicochemical Characteristics

Physicochemical analysis can provide useful information related to the quantification of the cheese’s basic components as well as its quality [15,16]. According to Lawrence et al. [17,18], pH, SM, FDM, and MNFS are important parameters regarding the composition of a cheese and can have a major effect on its quality.

The classification of cheeses is achieved in different countries according to various principles. Factors such as milk type, degree of ripening, method of manufacture, and physicochemical or microbiological characteristics are usually used for the grouping of a cheese variety [4,19]. In Greece, cheeses are classified primarily according to their moisture content. Specifically, the very hard cheeses have a maximum moisture of 32%, the hard have a maximum of 38%, the semi hard have a maximum of 46%, while the maximum moisture content of the soft cheeses must not exceed 58%. Fresh cheeses from milk, without ripening, contain less than 75% moisture and whey cheeses with or without ripening have less than 70% moisture content [20]. The classification of a cheese based on the moisture content provides information regarding its nutritional value and is widely used [4].

According to the Greek legislation, the term “quality” is accompanied with metric data on moisture and Fat-in-Dry-Matter content etc. [20]. Although cheeses with different production methods and characteristics were collected and analyzed in the present work, these cheeses were grouped together, primarily, according to their moisture content, as defined by the Greek legislation. In each group (soft, hard, etc.), the moisture and fat-in-dry-matter content was presented, which, according to the Greek legislation, was used to classify cheeses in qualities (very good, excellent, etc.) (Table 1, Table 2 and Table 3).

However, information on the label of the package or from the websites or from oral communication with the producer (when available) was also taken into consideration, in case needed. The general characteristics of Greek traditional cheese varieties have been previously studied [3,21,22,23,24,25]. In addition, the composition of commercial Greek cheese samples such as Kefalotyri, Anthotyros, Myzithra, Teleme, and Graviera [15,26,27,28,29,30] has previously been reported.

In the present study, 50 of the collected samples were classified as hard cheeses with a mean moisture of 33.8% and FDM of 49.9% (Table 4).

According to the Greek Codex Alimentarius [20], an excellent quality hard cheese has a moisture content < 35% and FDM > 47W; therefore, the hard cheeses of the present work could be classified as having an excellent quality. Kefalotyri and Graviera (excluding Graviera Kritis, Graviera Naxou, and Graviera Agrafon, which are PDO cheeses) are two well-known cheeses that are produced widely in Greece. Usually, the name of the local place where they are manufactured is used on the label of these cheeses. The composition of Kefalotyri cheese in the present study (Table 1) generally was in accordance with that found for commercial samples by Andrikopoulos et al. [28], while Graviera was in line with that of Vatavali et al. [29,30]. However, Zerfiridis et al. [31] observed a higher FDM for commercial Gruyere cheese samples than in this study. The values of fat of Pecorino of Ios Island were higher than those reported for Pecorino Romano and Sicilliano [32], whereas for the moisture and salt (Table 1) they were lower than that found by Kasapian et al. [15]. In general, these differences, when observed, may be attributed to variations in milk composition, manufacturing and storage conditions, degree of ripening, etc.

Six out of 102 cheeses belonged to the semi hard group, contributing to a mean moisture of 39.1% and FDM of 52.5% (Table 4). The cheeses of this group were of an excellent quality following Greek Codex Alimentarius [20], as an excellent quality semi hard cheese has a moisture content < 40% and FDM > 50%. The composition of semi hard Kashkaval cheese (Table 2) is generally comparable with those reported for pasta filata cheeses [23,33] and fulfill (dry matter of 60.8% and FDM of 49.2%, Table 2) the requirements (dry matter > 58%, FDM > 45%) of the EU [34]. Under this context, Kashkavali Pindou applied for a PDI indication [1].

Eighteen soft cheese samples were present in this study, with a mean moisture of 53.9% and FDM of 59.8% (Table 4). These cheeses were characterized as having an excellent quality (moisture of < 54%, FDM of > 46%) [20]. Teleme, a white brined cheese manufactured from sheep, goat, and cow milk or mixtures of them, had a generally similar composition to that determined by Andrikopoulos et al. [28] (Table 2). Touloumotyri had a higher moisture content than that found by Kasapian et al. [15] (Table 2). Kariki is a cheese that is matured in an empty gourd for 40 days up to seven months. This cheese, as well as Mpalaki soft of Tinos, had a low moisture content (37.4% and 36.9%, respectively) and could have been classified to the hard cheeses group, but both cheeses were characterized as soft, with a maximum moisture content of up to 55%, following the piece of information present on their label (Table 2). Tsalafouti is a spread cheese seeking registration as a PDO Greek cheese [35]. The composition of Tsalafouti cheese samples from a Greek market (Table 3) was generally in line with the data reported for industrially produced Tsalafouti cheese by Pappa and Kondyli [3]. The composition of whey cheeses is shown in Table 3 and Table 4. Different values were reported for a similar cheese produced in Turkey, namely Mud whey cheese [36]. Some differences were observed between the composition of Myzithra and Anthotyros cheeses of the present study and other commercially similar cheeses [25,28], possibly due to differences in their manufacturing process.

Although the cheeses studied here are very much appreciated by consumers locally, for several of them, there is a lack of scientific information and, to the best of our knowledge, the identity and the composition of some cheeses was studied for the first time. Such cheeses are Malathouni of Tinos, tyri of Mpournias, volaki of Andros and skotyri of Ios. Furthermore, Tyromalama, Galomyzithra, and Sitaka of Kasos Island (Table 2) were found to possess a very low salt content (0–1%) and can be suitable for consumers on a low sodium diet. However, in order to better establish their identity, more samples of each cheese need to be analyzed.

Sheep milk is widely preferred in cheesemaking as it results in the manufacture of a cheese with a higher yield than from cow. This is due to its higher protein, fat, and total solids content compared with cow milk [37,38]. In Greece, sheep and goat milk is mainly used for cheese production, while cow milk is mainly used for direct consumption, and IN the year 2020, 154,868 tonnes sheep, 36,495 tonnes goat, and 23,580 tonnes cow cheeses were produced [39]. In the present study, 37 samples were produced from the mixture of sheep and goat milk; 8 samples from solely sheep milk; 11 samples from goat milk; 9 samples from cow milk; 9 samples from mixture of sheep, goat, and cow milk; 1 sample from a mixture of sheep and cow milk; and 1 from other milk namely buffalo milk, while in 26 samples, the kind of milk used was not established because it was not reported on the package.

### 3.2. Discriminant Analysis

Discriminant analysis was applied to the 13 physicochemical cheese attributes to evaluate if the 100 samples could be distinguished by milk type. Two samples were excluded from discriminant analysis on the basis of milk because there was only a single sample per milk type group (buffalo milk or a mixture of sheep and cow milk). The milk type, cheese type, and location (cheese produced in a Greek island or in mainland) effects on composition and physicochemical characteristics of non-PDO/non-PGI cheeses for the present study are presented in Table 4. Neither location, nor milk type, had any effect on the physicochemical cheese attributes (*p* > 0.05), except from the color of goat cheese samples that were less yellow (lower b* values, *p* < 0.05) in comparison with the cheese samples made from cow milk. Color changes could be observed as an effect of diet, breed, lactation time, light exposure during processing, packaging, and distribution [40]. This difference in cheese color may reflect the milk color differences between goat and cows, which is attributed to their different carotenoid contents [41,42]. Bovines show a higher efficiency in the conversion of carotenoids, particularly β-carotene, into retinol in the enterocytes in comparison with ovines; therefore, cow milk is more yellow than goat milk [43]. In close agreement with the results of the present study, Lucas et al. [44] reported a β-carotene content of 3.78 mg/kg fat for 301 cow milk cheese samples, whereas the respective mean for 106 goat milk cheese samples was 0.00 mg/kg fat.

On the other hand, it was cheese type that had a significant effect on the composition and physicochemical characteristics (*p* < 0.05), except for redness (a*), which was unaffected (*p* > 0.05). Hard and semi hard cheeses showed higher protein, MNFS (*p* < 0.05) in comparison to soft, spread and whey cheeses. The fat and salt contents were shown to be higher in hard and semi hard cheeses compared with spread and whey cheese (*p* < 0.05). However, when fat was expressed on a dry matter basis, soft cheeses showed the greatest fat percentage, (59.8%), whereas hard and whey cheeses showed the least, at 49.9 and 45.6%, respectively (*p* < 0.05). The composition differences observed can explain color differences, namely more yellow (higher b* values) and less light color (decreased L* values) for hard cheeses compared with the rest of those in the present study (*p* < 0.05). The salt content differences observed were not altered when salt was expressed in the moisture content.

Cheeses exposed to natural and artificial light throughout processing, packaging, distribution, and at retailers are likely to be subjected to peroxidation. Light exposure causes the formation of off flavors, loss in nutritional value, and color changes, which rapidly impair product quality and marketability [11]. As a result, it is important to classify cheeses according to their susceptibility to oxidation and thus their market life. Cheese type had a significant effect on oxidative stability (*p* < 0.05). The lowest MDA values, indicative of an improved oxidative stability, were present in the hard and semi hard cheese samples, possibly due to their lower fat content on a dry matter basis compared with that for soft cheeses. However, because of the high SEM and the conservative Bonferroni adjustment method applied, no significant pairwise differences were detected. In general, the MDA values assessed in the present study were in accordance with those reported in previous studies [13,45,46]. In detail, the impact on the biochemical changes in cheese during ripening (proteolysis, lipolysis, and glycolysis) may influence its sensory characteristics. Under this context, proteolysis and lipolysis reactions had a significant important role in the sensory of Gouda-type cheese [45,46]. In Cheddar cheese, it was shown that the antioxidant activity of water-soluble extracts was correlated to the ripening period. More specifically, a decrease in antioxidant activity after the fifth month of ripening was reported, which might be indicative that the antioxidant peptides were not resistant to further proteolysis. It seems that proteolytic enzymes of adjunct cultures may contribute significantly to the increased production of peptides related to the antioxidant activity and sensory properties [47]. Similarly, in white brined goat milk cheese, the antioxidant activities of all of the examined samples increased until day 60, but then decreased at the end of the ripening period, indicating the role of using adjunct cultures in order to release bioactive peptides [48].

Differences in MDA values may be correlated not only with the type of cheese and its manufacture procedure, but also the milk type used during cheese making. In the present study, milk type did not have a significant effect on MDA values (*p* = 0.13), although higher values of MDA were noted in cheeses produced from sheep compared with cow milk (91.8 vs. 24.5%, Table 4). Nevertheless, studies examining milk showed that sheep milk contained a higher PUFA content (~25%) compared with cow milk, leading to a higher risk for lipid peroxidation and consequently to a higher MDA content. Indeed, the MDA content in sheep compared with cow milk was higher (13.40 vs. 8.07%) after 24h of storage at room temperature [49]. Moreover, the same researchers observed a strong positive correlation between PUFA and the index of lipid oxidation (MDA) [49]. Additionally, the total antioxidant capacity, determined using ORAC assay, of low-fat pasteurized milk was significantly lower (13.624–13.984 μmol TE/l) than that of milk with a higher fat content (14.124–14.216 μmol TE/l) [50]. A significant higher total antioxidant capacity, as determined by ABTS assay, was also found in cow milk with 3% fat than cow milk with 0.5–1.5% fat and skimmed milk [51].

**Figure 2:**
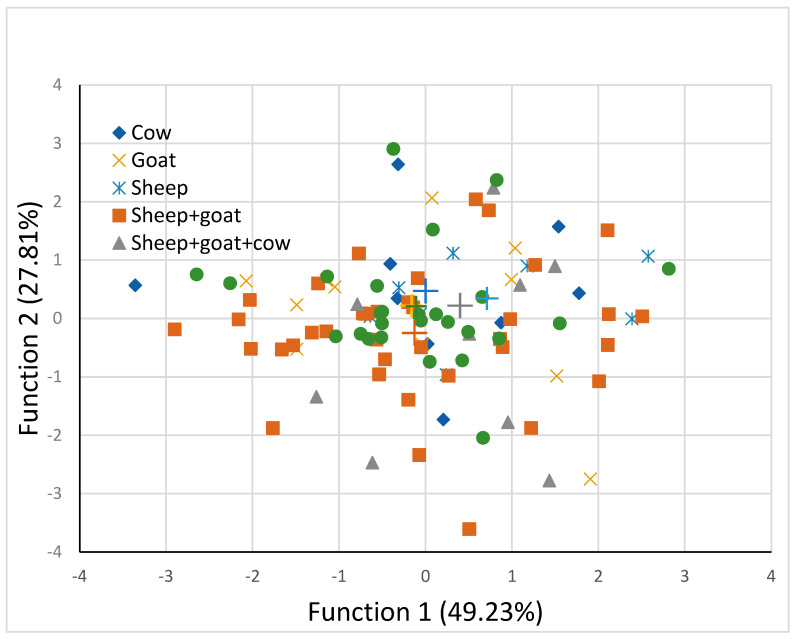
Discriminant analysis for different milk types using two discriminant functions of composition and physicochemical properties (+ indicates group centroid).

Two discriminant functions were found to be significant (*p* < 0.01) for distinguishing the samples among the different milk types. A graph of the two discriminant functions is shown in Figure 2.

The separation between milk type groups was not so clear and this was also evident by the close placement of the group centroids. As shown in Table 5, the sample percentage that was correctly classified into the appropriate milk type group was estimated to be low, at 62.8%.

Cow, goat, and sheep milk group samples were less misclassified, as 33.3, 18.2, and 25%, respectively, in comparison with their mixtures that were misclassified to a greater extent, as 49.6, 55.6 and 42.3%, for sheep and goat, sheep and goat and cow, and not defined milk groups, respectively. The stepwise discriminant analysis showed that 9 out of 13 characteristics were primary responsible for the discrimination of samples into milk-type groups. These elements were color attributes L, a and b, salt, ash, FDM, MNFS, SM, and MDA contents. A plausible explanation may be related to the differences in milk chemical composition between the three animal species, namely cow, sheep, and goat, and manufacture procedure and ripening. Sheep milk has been reported to be preferable by several cheese producers due its higher protein, fat, and total solids content compared with cow milk [37,38]. Under this scope, it should be note that previous work by our team on PDO cheeses [9] revealed that using physicochemical parameters and fatty acids during discriminant analysis, the percentage of the samples that were classified into the correct group according to the milk type, used for cheese manufacture, was 99.1%.

**Figure 3:**
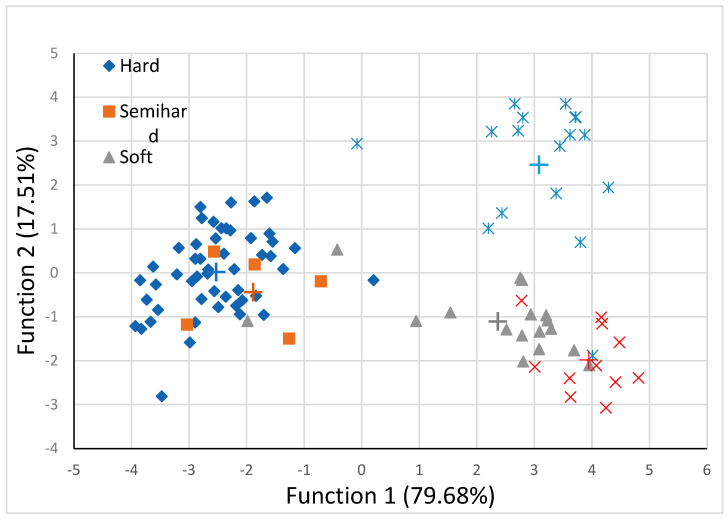
Discriminant analysis for different cheese types using two discriminant functions of composition and physicochemical properties (+ indicates group centroid).

The discriminant analysis of the physicochemical cheese attributes according to the type of cheese is presented in Figure 3.

There were three discriminant functions significant (P<0.01) for distinguishing the samples among the cheese-type groups. A plot of the first two discriminant functions is presented in Figure 3. A clear separation between hard, soft, spread, and whey cheeses was observed, whereas semi hard cheese samples were clustered into the hard cheese group, confirmed from the hard and semi hard group centroids that were placed close together. As shown in Table 6, 82.1% of the samples were correctly classified into the appropriate cheese type group, whereas a 17.9% were misclassified.

Hard cheese samples were misclassified as semi hard cheese at a rate of 14% (7 out of 50 samples), whereas one semi hard cheese sample was misclassified as hard cheese. Two soft cheese samples, out of 17, were misclassified as spread cheese and hard cheese, respectively. Two spread-type samples out of 11 were misclassified as soft-type cheeses, whereas the lowest rate of misclassification (11.8%) was observed for whey cheese samples. The stepwise discriminant analysis showed that 7 out of 13 characteristics were mainly responsible for the discrimination of samples into cheese-type groups. These elements were color attributes a and b, moisture, ash, fat, MNFS contents, and pH. A plausible explanation may be the differences in milk chemical composition between the three animal species, namely cows, sheep, and goats, and the manufacture procedure and ripening. Previously, using the total elemental fingerprint of PDO cheeses during discriminant analysis based on different milk types, 94.6% of cases were classified correctly [10]. A reasonable explanation might be connected to various parameters used at discriminant analysis (physicochemical, elements, and the fatty acids) or the difference between PDO and cheeses of limited production, such as those used in the present study. Differences between PDO and cheeses of limited production not listed in the EU Geographical Indications Registers indicate the potential benefits of a registration of an agricultural product. Potential benefits of a registration may be related to standardized production technology (type of milk used, specific details during manufacture, etc.) that provide high and constant quality and safety of a product [52]. As a certification of geographical indication is not applicable for those locally produced cheeses, the results of the present study can support and valorize local production. It is necessary to ensure that cheeses of limited productions are available to consumers all year round, as some of them are produced regionally and are hardly found throughout the country. Events, such as local festivals, agritourism, and regional celebrations, may be a way for manufacturers to promote their cheeses. On farm shops, e-commerce, local markets, and supermarkets may be appropriate actions to build a close relationship between purchaser and producer.

## 4. Conclusions

The consumer demand for buying locally produced cheeses of high quality is on the rise. Therefore, in the present work, the composition and the physiochemical characteristic of various non-PDO and non-PGI cheeses purchased from the Greek market were presented. Cheeses were grouped in different categories (hard, semi hard, soft, spread/processed, and whey). Milk and cheese types were correctly assigned for 62.8 and 82.1% of samples, respectively, through discriminant analysis. To the best of our knowledge, the characteristics of some cheeses such as Malathouni of Tinos, tyri of Mpournias, tyromalama, volaki of Andros, and galomyzithra and skotyri of Ios are reported for the first time. Generally, the cheeses of the present study were of a premium quality.

## Figures and Tables

**Table 1:** Composition and physicochemical characteristics of non-PDO and non-PGI hard cheeses purchased from the Greek market.

Cheese	n		pH	Moisture %	Fat %	Salt %	Protein %	Ash %	MNFS %	FDM %	SM %	MDA (ng/g)	L*	a*	b*
**Hard cheeses**															
Graviera	18	Mean	5.2	35.6	32.2	2.1	25.5	4.9	52.6	50	5.9	38	82.5	−0.08	25.8
		S.E.	0.1	0.6	0.7	0.1	0.8	0.2	0.7	1	0.3	6	0.8	0.41	0.7
Kefalotyri	17	Mean	5.3	36.8	31.1	3.1	24.8	5.7	53.5	49.2	8.3	55	84.1	−1.0	24.2
		S.E.	0.1	0.5	0.6	0.1	0.7	0.2	0.6	0.8	0.5	6	0.6	0.4	0.5
Manoura	3	Mean	5.1	35	31	2.8	25	5	50	47.4	9	9	86.5	1	21.7
		S.E.	0.1	4	2	0.9	1	1	4	0.7	2	2	0.9	0.5	0.7
Oil cheese ^1^	3	Mean	5.4	34	36	2.0	23	4.7	52	53.4	6.1	5 × 10	83.2		25
		S.E.	0.3	3	2	0.2	1	0.1	4	0.6	0.4	3 × 10	0.9		1
Melichloro of Lemnos Island	2	Mean	4.9	35	35	1.6	21	3.8	53	53	4.60	7 × 10	88	0	22
		S.E.	0.1	1	5	0.1	3	0.3	1	6	0.01	6 × 10	2	-	2
Malathouni of Tinos Island	1		4.2	22.1	15.0	1.4	24.3	1.9	26	19.3	6.5	26.1	85.9	−0.06	25.7
Pekorino of Ios Island	1		5.4	28.9	37.8	1.7	25.9	4.2	46.4	53.1	5.7	175.0	81.9	0.27	26.7
Saganaki	1		5.2	36.0	35.3	2.7	23.5	5.4	55.5	55.0	7.5	131.2	83.6	−0.32	24.1
Smoked cheese ^2^	1		3.3	34.5	31.5	2.7	27	5.2	50.4	48.1	7.7	8.6	83.1	−0.38	28.8
Tyri of Mpournias-Andros Island	1		5.3	25.3	42.0	2.2	28.4	3.2	43.7	56.3	8.6	4.6	75.5	6.1	32.3
Other hard cheeses ^3^	2	Mean	5.5	32	32	2.7	3	5.3	48.3	47	8.2	15	85	0	24
		S.E.	0.2	2	5	0.4	1	0.4	0.9	6	0.8	6	2	-	3

MNFS: moisture-in-non-fat substance; FDM: fat-in-dry matter; SM: salt-in-moisture; MDA: malondialdehyde (ng/g); L* = lightness; a* = redness; b* = yellowness. No standard error is available for cheeses with n=1. ^1^ Oil cheese from various regions of Greece including Kefalonia Island, Zakynthos Island, and Milos Island. ^2^ Smoked hard cheese from the Ioannina region. ^3^ Hard cheeses from Naxos Island and near the city of Lamia.

**Table 2:** Composition and physicochemical characteristics of non-PDO and non-PGI semihard and soft cheeses purchased from the Greek market.

Cheese	n		pH	Moisture %	Fat %	Salt %	Protein %	Ash %	MNFS %	FDM %	SM %	MDA (ng/g)	L*	a*	b*
**Semihard**															
Semihard	2	Mean	4.9	41.9	32	1.7	24.3	4.4	62	56	4.1	29	85	1.2	27.0
		S.E.	0.1	0.3	5	0.3	0.1	0.4	5	9	0.7	4	1	0.1	0.5
Kashkaval	2	Mean	4.9	39	30	1.9	25	4.4	56	49	5	2	84	−1.3	24.7
		S.E.	0.1	4	3	0.6	2	0.5	4	1	2	1	1	0.2	0.5
Tyromalama	1		5.4	39.9	31.3	0.0	24.5	3.1	58.1	52.0	0.0	25.6	80.9	−2.1	25.1
Volaki of Andros Island	1		5.1	43.6	30.3	1.7	21.5	3.1	62.5	53.6	3.8	15.1	89.9	1.8	25.5
**Soft**															
Brined soft ^1^	10	Mean	4.3	55	27	2.2	14.8	3.2	75	60	4.0	9	91.5	−1.0	12.4
		S.E.	0.1	1	1	0.2	0.4	0.3	2	3	0.3	2	0.7	0.1	0.5
Mpalaki soft of Tinos Island	1		4.9	36.9	33.0	1.5	25.3	2.1	55.1	52.3	3.9	43.9	86.2	1.1	26.1
Kariki of Tinos Island	1		5.6	37.4	40.8	2.4	19.3	2.3	63.2	65.2	6.42	11.1	72.0	1.0	21.1
Galomyzithra	1		4.5	58.1	31.5	1.0	8.0	1.6	84.8	75.1	1.8	33.2	91.3	−0.93	14.4
Xinotyri soft of Mykonos Island	1		4.7	52.4	25.0	2.2	17.7	2.6	69.9	52.5	4.2	20.3	91.7	−1.24	17.1
Petroti of Mykonos Island	1		4.8	62.9	17.8	1.5	13.7	1.9	76.4	47.9	2.3	9.9	95.9	−1.4	15.2
Sitaka of Kasos Island	1		5.4	58.1	33.0	0.1	14.0	2.0	86.7	78.8	0.17	4.6	73.3	9.2	34.2
Telemes	1		4.5	52.8	25.3	1.5	16.9	2.5	70.6	53.5	2.7	44.3	89.1	0.16	20.6
Touloumotyri of Karistos Island	1		5.1	53.6	31.3	3.2	16.5	5.0	78.0	67.4	6.0	38.1	88.0	−0.50	15.2

MNFS: moisture-in-non-fat substance; FDM: fat-in-dry matter; SM: salt-in-moisture; MDA: malondialdehyde (ng/g); L* = lightness; a* = redness; b* = yellowness. No standard error is available for cheeses with n = 1. ^1^ Sheep and goat brine cheese from Corfu Island; sheep and goat cheese with chili, buffalo cheese; and goat cheese in brine from various regions of Greece including Ioannina, Arta, Corfu Island, and a region near Larissa.

**Table 3:** Composition and physicochemical characteristics for non-PDO and non-PGI of spread/processed and whey cheeses purchased from the Greek market.

Cheese	n		pH	Moisture %	Fat %	Salt %	Protein %	Ash %	MNFS %	FDM %	SM %	MDA (ng/g)	L*	a*	b*
**Spread/processed**															
Spread cheese ^1^	4	Mean	4.5	71	13	1.1	11	1.6	81	43	1.5	16 × 10	93.7	−1.1	15.2
		S.E.	0.2	5	4	0.2	2	0.1	2	5	0.2	5 × 10	0.7	0.2	0.4
Tsalafouti	3	Mean	4.1	71	14	1.2	10	1.6	82.4	47	1.6	8 × 10	94.3	−0.97	12.0
		S.E.	0.1	2	2	0.1	1	0.1	0.8	3	0.1	3 × 10	0.5	0.08	0.7
Skotyri of Ios Island-processed	2	Mean	4.0	59	30	1.9	11.6	2.6	84	72	3.2	21	83.8	0.3	14.3
		S.E.	0.1	2	6	0.1	0.3	0.1	5	1	0.3	2	0.6	0.1	0.2
Xinomyzithra of Kimolos Island	1		4.1	61.5	21.8	0.4	14.2	0.8	78.6	56.5	0.57	23.3	88.2	−0.46	15.6
Other processed ^2^	1		4.6	66.7	15.5	1.6	11.0	1.9	79.0	46.6	2.3	54.1	86.9	−1.53	11.6
**Whey cheeses**															
Anthotyros	4	Mean	6.1	71	13	0.6	12	1.3	81.7	44	0.8	9	87.9	−1.4	16.5
		S.E.	0.3	2	2	0.2	1	0.3	0.8	5	0.2	3	0.9	0.3	0.8
Anthotyros partly skimmed	3	Mean	6.7	71	12	0.4	12	1.4	81.7	44	0.5	6.7	88.8	−1.7	17.2
		S.E.	0.1	3	3	0.2	1	0.9	0.8	7	0.1	0.5	0.6	0.3	0.6
Dry myzithra partly skimmed	3	Mean	5.4	5	13	4	26	7	57	2 × 10	8	14 × 10	91.4	−0.4	13.2
		S.E.	0.1	1	5	1	7	1	4	1 × 10	2	5 × 10	0.2	0.2	0.5
Fresh myzithra partly skimmed	2	Mean	5.8	66	17	0.90	11.8	1.7	80	50.1	1.3	4 × 10	89.3	−1.6	1.6 × 10
		S.E.	0.2	3	2	0.04	0.1	0.0	2	0.1	0.0	2 × 10	0.6	0.1	0.1 × 10
Myzithra	2	Mean	5	71	14	0	15	1.7	82	47	0	28	90	−1.1	14
		S.E.	1	4	3	−	5	0.6	2	4	−	1	2	0.0	2
Dry anthotyros	1		6.4	37.5	38.6	2.6	17.3	3.8	61.0	61.6	6.7	205.9	88.5	−0.51	17.2
Dry myzithra	1		6.0	50.8	13.3	4.7	20.5	9.2	58.5	26.9	9.2	30.4	90.1	−0.49	13.6
Other whey cheeses ^3^	1		6.0	45.9	50.5	0.9	8.0	1.3	92.6	93.3	1.8	114.8	92.3	−1.4	14.6
*p*-value			<0.001	<0.001	<0.001	<0.001	<0.001	<0.001	<0.001	<0.001	<0.001	<0.001	<0.001	<0.001	<0.001

MNFS: moisture-in-non-fat substance; FDM: fat-in-dry matter; SM: salt-in-moisture; MDA: malondialdehyde (ng/g); L* = lightness; a* = redness; b* = yellowness. No standard error is available for cheeses with n = 1. ^1^ Spread cheese manufactured near the cities of Ioannina and Lamia. ^2^ Manufactured near the city of Arta. ^3^ Produced near the city of Arta.

**Table 4:** The effect of location, type of milk, and type of cheese on the composition and physicochemical characteristics of non-PDO and non-PGI cheeses from the Greek market (L.S.M. ± S.E.).

Factor		n	pH	Moisture %	Fat %	Salt %	Protein %	Ash %	MNFS %	FDM %	SM %	MDA (ng/g)	L*	a*	b*
Location	Mainland	58	5.0	51.8	24.5	1.8	18.2	3.4	67.8	49.8	3.9	52.7	88.1	−0.63	19.4
	Island	44	4.8	49.2	27.3	1.4	17.8	2.8	67.0	54.4	3.2	52.5	87.4	−0.04	18.9
	S.E.		0.1	1.6	1.5	0.2	1.0	0.3	1.7	2.4	0.6	12.8	0.9	0.39	0.8
	*p*-value		0.099	0.096	0.068	0.13	0.648	0.084	0.594	0.05	0.22	0.989	0.386	0.117	0.485
Milk type	Sheep and goat	37	4.9	50.9	27.1	2.1	18.4	3.5	69.4	54.7	4.5	67.8	88.0	−0.61	18.8 ^ab^
	Goat	11	4.9	50.7	25.8	1.5	18.3	3.0	67.8	53.0	3.0	39.9	90.2	−0.75	15.4 ^b^
	Sheep, goat, and cow	9	5.2	53.0	26.5	1.6	17.7	3.3	71.5	56.3	3.5	22.9	85.8	−0.03	20.1 ^ab^
	Cow	9	5.0	48.1	27.1	1.5	29.8	2.6	64.5	50.9	3.8	24.5	85.4	0.77	22.5 ^a^
	Sheep	8	4.8	53.8	24.9	1.5	15.9	3.3	70.0	53.2	3.0	91.8	85.3	−1.4	19.0 ^ab^
	Sheep and cow	1	4.8	51.7	23.0	1.4	17.5	3.0	66.7	48.1	3.0	70.3	87.0	1.40	20.7 ^ab^
	Buffalo	1	4.5	43.0	30.7	1.4	18.0	2.9	61.7	53.9	3.5	44.6	92.2	−1.61	17.2 ^ab^
	Not defined	26	5.1	52.7	22.1	1.7	18.4	3.5	66.6	45.9	4.1	59.0	88.2	−0.48	19.4 ^ab^
	S.E.		0.1	3.3	3.1	0.5	2.0	0.7	3.4	4.9	1.1	25.6	1.8	0.79	1.5
	*p*-value		0.602	0.613	0.196	0.667	0.858	0.869	0.333	0.083	0.677	0.13	0.069	0.243	0.005
Cheese type	Hard	50	5.2 ^a^	33.8 ^a^	32.7 ^a^	2.2 ^a^	24.9 ^a^	4.7 ^a^	50.5 ^a^	49.9 ^a^	6.5 ^a^	38.0	83.7 ^a^	0.05	25.0 ^a^
	Soft	18	4.6 ^bc^	53.9 ^b^	27.5 ^a^	1.9 ^ab^	14.9 ^b^	3.0 ^bc^	74.4 ^b^	59.8 ^b^	3.6 ^b^	67.2	88.5 ^b^	−0.15	16.6 ^b^
	Whey	17	5.8 ^d^	60.5 ^bc^	18.3 ^b^	1.4 ^b^	14.8 ^b^	2.7 ^bc^	74.2 ^b^	45.6 ^a^	2.5 ^b^	52.7	89.5 ^b^	−1.0	15.1 ^b^
	Spread	11	4.1 ^c^	65.2 ^c^	19.1 ^b^	1.0 ^b^	10.9 ^b^	1.4 ^c^	80.2 ^b^	52.6 ^ab^	1.5 ^b^	90.5	90.7 ^b^	−0.4	14.3 ^b^
	Semihard	6	5.0 ^ab^	39.1 ^a^	31.8 ^a^	1.4 ^ab^	24.5 ^a^	3.9 ^ab^	57.7 ^a^	52.5 ^ab^	3.7 ^ab^	14.6	86.3 ^ab^	−0.15	24.7 ^a^
	S.E.		0.1	2.4	2.2	0.3	1.4	0.5	2.5	3.5	0.8	18.4	1.3	0.57	1.1
	*p*-value		<0.001	<0.001	<0.001	0.002	<0.001	<0.001	<0.001	0.012	<0.001	0.026	<0.001	0.293	<0.001

MNFS: moisture-in-non-fat substance; FDM: fat-in-dry matter; SM: salt-in-moisture; MDA: malondialdehyde (ng/g); L* = lightness; a* = redness; b* = yellowness. ^a–d^ Data with different superscripts, within a column, differ significantly (*p* < 0.05).

**Table 5:** Classification table for different milk types based on the composition and physicochemical characteristics of cheeses through discriminant analysis.

		Predicted Milk Type					
Actual Milk Type	Group Size	Cow	Goat	Sheep	Sheep & Goat	Sheep, Goat & Cow	Not Defined
Sheep and Goat	37	4	3	4	19	3	4
		(10.8%)	(8.11%)	(10.81%)	(51.4%)	(8.11%)	(10.8%)
Goat	11	1	9	1	0	0	0
		(9.1%)	(81.8%)	(9.1%)	(0.0%)	(0.0%)	(0.0%)
Sheep, Goat, and Cow	9	1	1	2	0	4	1
		(11.1%)	(11.1%)	(22.2%)	(0.0%)	(44.4%)	(11.1%)
Cow	9	6	1	0	0	2	0
		(66.7%)	(11.1%)	(0.0%)	(0.0%)	(22.2%)	(0.0%)
Sheep	8	0	0	6	1	0	1
		(0.0%)	(0.0%)	(75%)	(12.5)	(0.0%)	(12.5%)
Not defined	26	2	3	2	3	1	15
		(7.7%)	(11.5%)	(7.8%)	(11.5%)	(3.9%)	(57.7%)

Cases correctly classified: 62.8%.

**Table 6:** Classification table for different cheese types based on the composition and physicochemical characteristics of each cheese through discriminant analysis.

		Predicted Cheese Type				
**Actual Cheese Type**	Group Size	Hard	Semi-Hard	Soft	Spread	Whey
Hard	50	42	7	1	0	0
		(84.0%)	(14.0%)	(2.0%)	(0.0%)	(0.0%)
Soft	17	2	0	13	2	0
		(11.8%)	(0.0%)	(76.5%)	(11.8%)	(0.0%)
Whey	17	0	0	1	1	15
		(0.0%)	(0.0%)	(5.9%)	(5.9%)	(88.2%)
Spread	11	0	0	2	9	0
		(0.0%)	(0.0%)	(18.2%)	(81.8%)	(0.0%)
Semihard	5	1	4	0	0	0
		(20.0%)	(80.0%)	(0.0%)	(0.0%)	(0.0%)

Cases correctly classified: 82.1%.

## Data Availability

Data is contained within the article.
